# A GRX-SOD module maintains superoxide homeostasis and meristem development in maize

**DOI:** 10.1007/s44307-026-00133-8

**Published:** 2026-09-04

**Authors:** Ting Guo, Xintong Liu, Ruoshu Yang, Yajie Wang, Fang Yang

**Affiliations:** 1 State Key Laboratory of Biocontrol, Guangdong Provincial Key Laboratory of Plant Stress Biology, School of Agriculture and Biotechnology, Sun Yat-Sen University, Shenzhen, 518107 China; 2 National Key Laboratory of Crop Genetic Improvement, Huazhong Agricultural University, Wuhan, 430070 China

**Keywords:** Maize, Shoot apical meristem, Glutaredoxin, Superoxide dismutase, Reactive oxygen species

## Abstract

The continuous initiation of plant aerial organs relies on the maintenance of dynamic stem cell homeostasis within the shoot apical meristem (SAM). Although reactive oxygen species (ROS) have emerged as crucial signals determining cell fate, how plants precisely maintain SAM-specific ROS homeostasis remains to be elucidated. Here, we report that a redox regulatory module comprising the maize CC-type glutaredoxin MSCA1 and its homologs regulates meristem architecture by maintaining superoxide homeostasis in the SAM. We found that the superoxide anion (*O*_2_^•−^) is specifically enriched in the central zone of the maize SAM, whereas the loss of the GRX module leads to strikingly decreased *O*_2_^•−^ accumulation, resulting in SAM size reduction and impaired leaf formation. MSCA1 physically interacts with the superoxide dismutase ZmCSD5, and loss of GRX function is associated with elevated total SOD activity, supporting a working model in which MSCA1 may restrain ZmCSD5-associated SOD activity and thereby limit excessive *O*_2_^•−^ scavenging in the SAM. Furthermore, loss-of-function *zmcsd5* mutants exhibit a significantly enlarged SAM, genetically supporting the proposed MSCA1-ZmCSD5 regulatory module. Our findings reveal how superoxide homeostasis is precisely maintained in the SAM to control development, providing potential genetic targets for the molecular improvement of crop architecture and yield traits.

**Supplementary Information:**

The online version contains supplementary material available at https://doi.org/10.1007/s44307-026-00133-8.

## Introduction

The initiation and development of all above-ground organs in plants depend on the dynamic homeostasis of the stem cell niche in the shoot apical meristem (SAM) (Barton 2010; Dresselhaus et al. 2025; Heidstra and Sabatini 2014). Previous studies have revealed the critical roles of complex transcriptional and signaling networks, primarily the classical CLAVATA-WUSCHEL pathway (Mayer et al. 1998; Schoof et al. 2000) and its post-translational modification regulation such as the DA1 peptidase (Cui et al. 2024), in maintaining meristem homeostasis (Demesa-Arevalo et al. 2024). Recent studies have indicated that the SAM is also embedded within an exquisite metabolic microenvironment. For example, the apical hypoxic niche of the SAM, shaped jointly by morphological features and local metabolism, can act as a spatial positional signal to participate in regulating stem cell activity and coordinating the pace of organogenesis (Voloboeva et al. 2026; Weits et al. 2019). However, within this specific meristematic microenvironment, how basal cellular metabolism, particularly represented by the redox state, acts as a signaling instruction to directly dictate stem cell fate remains a scientific question awaiting in-depth elucidation.

In recent years, reactive oxygen species (ROS) have been redefined from traditional metabolic byproducts to critical signaling molecules that determine cell developmental fate and coordinate stress responses in both animals and plants (Considine and Foyer 2014; Mittler 2017; Zeng et al. 2024). In the plant shoot apical meristem, different types of ROS signals exhibit specific spatial distributions and exert antagonistic regulatory functions. Studies in the model plant Arabidopsis have shown that the superoxide anion (*O*_2_^•−^) is highly enriched in the CZ of the SAM to maintain stem cell identity, and can determine cell fate by mediating epigenetic modifications such as DNA demethylation of target genes (Wang et al. 2025; Zeng et al. 2023); whereas hydrogen peroxide (*H*_*2*_*O*_*2*_) is mainly distributed in the PZ to promote cell differentiation (Zeng et al. 2017). This regulatory mode based on the specific spatial distribution of ROS exists not only in above-ground parts but is also highly conserved in the root apical meristem (e.g., ROS signaling mediated by the UPB1 transcription factor and RGF1) (Tsukagoshi et al. 2010; Yamada et al. 2020). Furthermore, dynamic changes in local ROS signals can prolong stem cell thermotolerance and maintain homeostasis under extreme environments (e.g., heat stress) by inducing reversible protein phase separation or modulating mRNA translation efficiency (Huang et al. 2021, 2025; Liu et al. 2024). To precisely maintain this fate-determining, stem cell-specific spatial distribution of ROS, plants must exert strict spatiotemporal restrictions on ROS generation and scavenging within the microenvironment. However, the specific regulatory mechanisms underlying this stem cell-specific ROS homeostasis in plants, especially in monocot staple crops such as maize, remain largely unknown.

Glutaredoxins (GRXs), a class of small oxidoreductases ubiquitous in eukaryotes, play a core role in plant development and environmental adaptation by catalyzing the redox state of specific cysteine residues on target proteins (Meyer et al. 2009; Rouhier 2010; Wu et al. 2017; Zeng et al. 2017). In maize, the CC-type glutaredoxin MSCA1 and its two paralogs, ZmGRX2 and ZmGRX5, have been demonstrated to be key regulators of inflorescence meristem development, significantly modulating SAM size and plant architecture (Yang et al. 2015). Our previous study further revealed that MSCA1 can alter the DNA-binding activity of the bZIP transcription factor FEA4 via redox modification, thereby regulating inflorescence meristem development (Yang et al. 2021). However, whether MSCA1, functioning as an oxidoreductase, directly acts on the basal ROS metabolic network to participate in the maintenance of ROS homeostasis in the meristem remains unknown.

Here, we unveil a novel molecular mechanism underlying superoxide homeostasis and meristem development, in which maize MSCA1 and its paralogs (collectively referred to as GRXs) maintain superoxide homeostasis in SAM, particularly the spatial distribution pattern of the superoxide anion, by directly regulating the activity of ROS metabolic enzymes. This study elucidates the critical role of GRX-regulated superoxide homeostasis within the central zone stem cell niche in determining SAM architecture, and provides a molecular basis for optimizing meristem activity and plant architecture.

## Materials and methods

### Plant materials

The CRISPR/Cas9 knockout allele *zmcsd5* was generated in B73 inbred line and backcrossed twice to standard B73 to remove Cas9. Mutant lines *msca1*, *zmgrx2*, *zmgrx5* and *grx* triple were described previously (Yang et al. 2021). These materials are all in a B73 background. All maize plants were cultivated in the greenhouse at Sun Yat-sen University (Shenzhen, Guangdong, China) under standard conditions. Phenotypic data and tissue samples were collected from seedlings at approximately 14 days after germination (DAG). The gRNA sequences and genetic markers for genotyping are provided in Table S1.

### Meristem measurement

For shoot apical meristem (SAM) measurement, shoot tips were dissected from 14-DAG seedlings and fixed in FAA solution (10% formaldehyde, 45% ethanol and 5% acetic acid) overnight at 4℃. Samples were dehydrated through a graded ethanol series and then cleared sequentially in 50% and 100% methyl salicylate for 1 h at each step. Cleared samples were visualized using a Nikon ECLIPSE Ni-U microscope. SAM size was quantified according to the geometric modeling method described previously (Yang et al. 2021).

### RNA in situhybridization

Shoot tips from 14-DAG seedlings from B73 were fixed and processed for RNA in situ hybridization as previously described (Greb et al. 2003). Templates for riboprobes included the full-length coding sequence (CDS) and portions of the 3'-untranslated region (3'-UTR) of *MSCA1*, *ZmGRX2*, *ZmGRX5* and *ZmCSD5*. Digoxigenin-labeled antisense RNA probes were synthesized using an in vitro transcription kit (Roche) following the manufacturer’s protocol. All primers used for template amplification are listed in Table S1. Images were captured using a Nikon ECLIPSE Ni-U microscope.

### Yeast Two-Hybrid (Y2H) assay

The full-length CDS of MSCA1 and CSD5/MSD2, including various mutated versions, were cloned into the bait vector pGBKT7 and the prey vector pGADT7. Interaction assays were performed by co-transforming the respective plasmids into the yeast strain Y2HGold (Clontech). Transformants were selected on double-dropout medium (SD/-Leu/-Trp) and screened for interactions on quadruple-dropout medium (SD/-Ade/-His/-Leu/-Trp). To verify protein expression, yeast total protein was extracted, and fusion proteins were detected via immunoblotting using rabbit anti-HA (Proteintech, 1:3,000) and mouse anti-Myc (ABclonal, 1:2,000) antibodies.

### BiFC and LCI assay

For the LCI assays, full-length CDS of MSCA1 and CSD5/MSD2 were cloned into JW771 (nLUC) and JW772 (cLUC), respectively, and transformed into *Agrobacterium* GV3101. For the BiFC assays, full-length CDS of MSCA1 was fused with the N-terminus of yellow fluorescent protein (YFP) of the vector pXY106, and the full-length CDS of CSD5/MSD2 was fused with the C-terminus of YFP of the vector pXY104. Cultures were resuspended in infiltration buffer (10 mM MES-K^+^, 10 mM MgCl2 and 100 μM acetosyringone, pH 5.6) to an OD_600_ of 0.8 and infiltrated into 3-week-old *Nicotiana benthamiana* leaves. At 48–60 h post-infiltration, leaves were treated with 1 mM luciferin (Promega), and luciferase signals were captured using a Lumazone Pylon2048B imaging system. Protein expression was confirmed by western blot using a goat anti-firefly luciferase antibody (Abcam, 1:2,000).

### Superoxide staining

Nitroblue tetrazolium (NBT) was used to visualize the superoxide-associated signal (Hückelhoven et al. 2000; Jiao et al. 2011). Shoot tips were infiltrated with 1 mg/ml NBT (Sigma-Aldrich) in 50 mM KH_2_PO_4_ (pH 7.6), and incubated in the dark for 12 h. Staining was terminated by transferring into boiling ethanol solution (ethanol:glycerin:glacial acetic acid = 3:1:1). The plants were then fixed and embedded as previously described (Andersen et al. 2008; Weigel and Jürgens 2002). The NBT staining intensity within the anatomically defined central zone was quantified using ImageJ. For pharmacological perturbation, 7-DAG seedlings were treated with 30 mM n-propyl gallate (PG) or 10 mM N, N'-dimethylthiourea (DMTU) until 14 DAG, when SAM size was measured. Because PG and DMTU are broad-spectrum radical scavengers rather than superoxide-specific reagents, these treatments were interpreted as perturbations of the redox environment rather than as superoxide-specific depletion.

### SOD activity measurement assays

The total SOD activity of SAM was measured using the SOD activity assay kit (Solarbio, BC5165, China) following the manufacturer's protocol with slight modification. Briefly, SAM tissues were homogenized in extraction buffer at a ratio of 0.1 g tissue per 1 mL buffer and centrifuged at 8000 g for 10 min at 4 °C. 20 μL of supernatant was added to the reaction mixture to a final volume of 200 μL. After incubation at 37 °C for 30 min, absorbance was measured at 450 nm. SOD inhibition rate and enzyme activity were calculated according to the kit formulas. Three biological replicates were analyzed.

### RNA-seq and data analysis

SAM tissue was collected from 14-DAG seedlings, with two biological replicates for each genotype, and immediately frozen in liquid nitrogen. Total RNA was extracted using RNA extraction kit (Genestonebio, TR205, China), and libraries were constructed using Illumina Standard Library Preparation Kit. The quality of raw data was controlled using FASTP v1.0.1. The clean reads were mapped to the maize reference genome (*Zea mays* AGPv4) using HISAT2 (v2.2.0) with default settings. StringTie (v2.2.3) was used to measure the abundance of RNA and DESeq2 was employed for differential expression analysis between two comparison groups by setting criteria for differentially expressed genes: |log_2_FoldChange|> 1 and *P*-value < 0.05. Gene Ontology (GO) enrichment was conducted using AgriGO v2.0 (http://systemsbiology.cau.edu.cn/agriGOv2/).

## Results

**Fig. 1:**
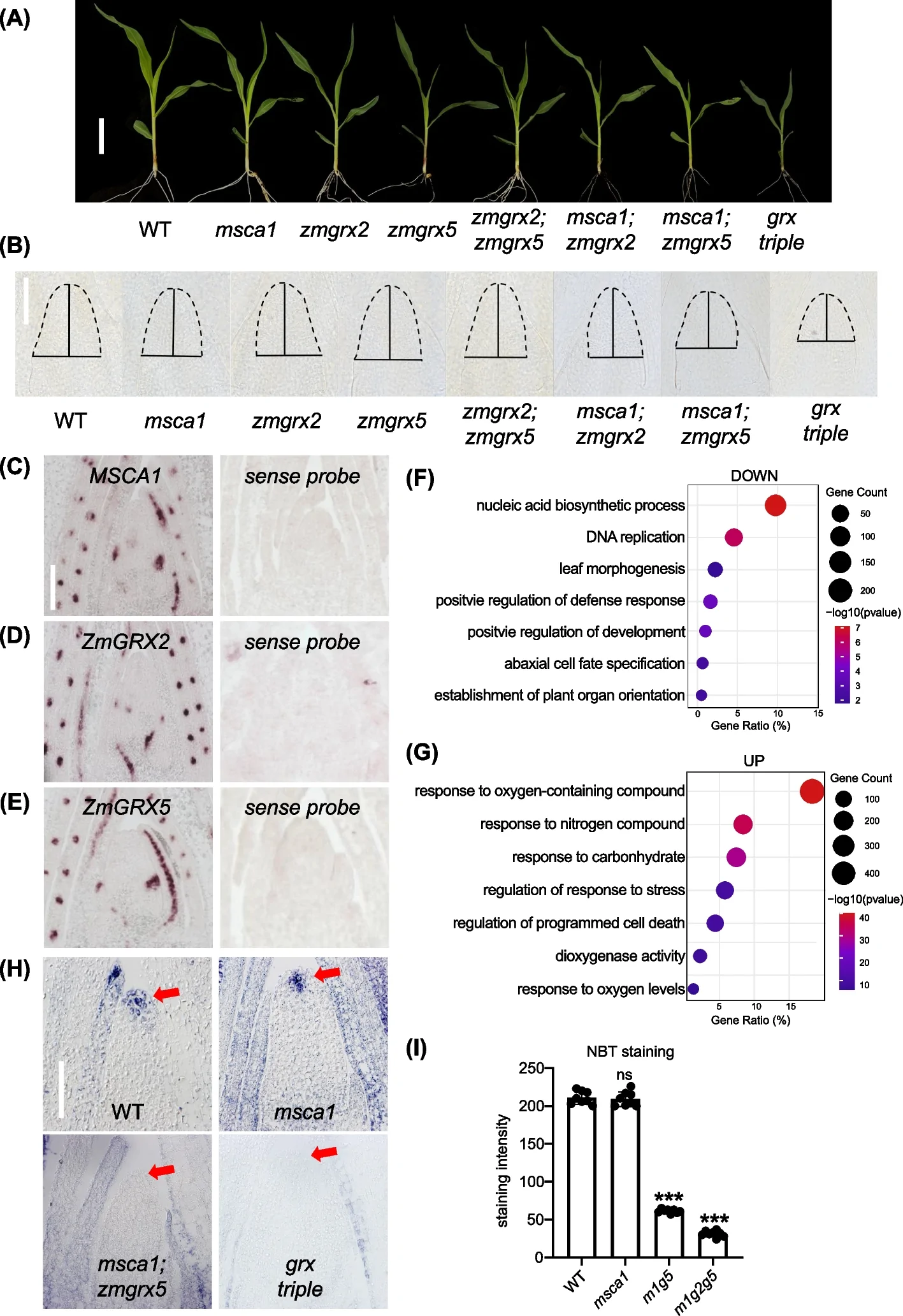
**A** Compared with WT, the *msca1; zmgrx5* double mutants and the *grx* triple mutant exhibit significantly reduced plant height. Bar = 5 cm. **B** Mutations in the three *GRX* genes result in a progressively reduced SAM volume. Representative images of cleared SAM from WT and the indicated mutants. SAM height and diameter marked by solid lines. Bar = 100 μm. **C-E** Expression patterns of *GRX* genes. RNA in situ hybridization using antisense probes for *MSCA1*, *ZmGRX2*, and *ZmGRX5* revealed strikingly similar and overlapping expression domains. All three genes are specifically expressed at leaf primordia initiation sites and within the vascular tissues of young leaves. Bar = 100 μm. **F-G** Gene Ontology analysis of biological pathways enriched in the mutant lines based on the downregulated (lower panel) (**F**) or upregulated (upper panel) (**G**) genes. **H-I** Representative NBT staining showing the spatial distribution of the superoxide-associated signal in the SAM (**H**). The central zone is indicated by red arrows. Bar = 100 μm. Quantification of NBT staining intensity within the central zone (**I**). Data are presented as mean ± SD (n = 8 biologically independent plants). Statistical significance was assessed using one-way ANOVA followed by Dunnett’s multiple-comparison test, with WT as the control group. ns, not significant; ***P < 0.001

Previous studies have reported that maize *MSCA1*, together with its two paralogs *ZmGRX2* and *ZmGRX5* (collectively referred to as three GRXs), regulates meristem size and coordinates plant architecture (Yang et al. 2015, 2021). To elucidate the genetic roles and further explore the mechanisms of these three *GRX* genes in early SAM development, we characterized the SAM size and plant architecture of various *grx* mutants at 14 days after germination (DAG). Compared to wild-type (WT), *msca1;zmgrx5* double and *msca1;zmgrx2;zmgrx5* triple mutants (hereafter referred to as the *grx* triple mutant) exhibited significantly reduced plant height (Fig. 1A, Figure S1A-B). Furthermore, we observed that SAM width and height were progressively reduced in *msca1* single, *msca1;zmgrx5* double, and *grx* triple mutants (Fig. 1B, Figure S1C-D). This indicates that three *GRX* genes redundantly promote meristem development, with *MSCA1* and *ZmGRX5* playing major roles. RNA in situ hybridization further revealed that three *GRX* genes were specifically expressed at leaf primordia initiation sites and in vascular tissues of developing leaves (Fig. 1C-E, Figure S1E). These data demonstrate that these three *GRX* genes play essential and redundant roles in meristem maintenance and leaf initiation, with *MSCA1* and *ZmGRX5* acting as the primary regulators.

**Fig. 2:**
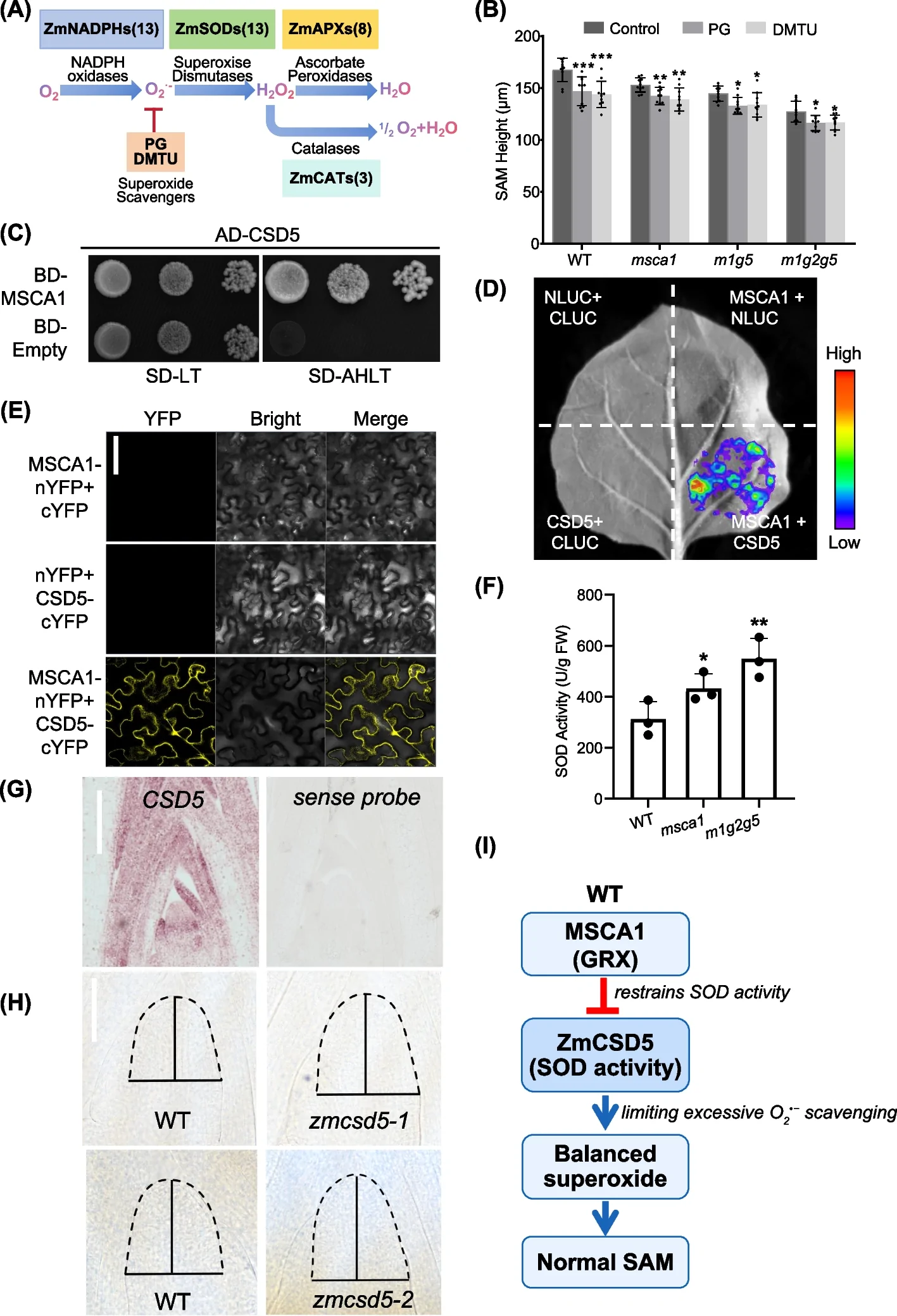
**A** A simplified schematic of the major ROS metabolic network in maize. **B** Quantitative morphometric analysis of SAM size in WT and mutants. Seedlings were treated with 30 mM PG or 10 mM DMTU. Data are presented as mean ± SD (*n* = 10 biologically independent plants). Statistical significance was assessed using two-way ANOVA with genotype and treatment as factors, followed by Šidák’s multiple-comparison test for comparisons of PG or DMTU treatment with the corresponding control within each genotype. *P < 0.05; **P < 0.01; ***P < 0.001. **C** Yeast two-hybrid (Y2H) assays demonstrating the interaction of MSCA1 with ZmCSD5. **D** LCI confirmed the interaction of MSCA1 with ZmCSD5. **E** BiFC analysis of the interaction between MSCA1 and ZmCSD5 in tobacco leaves. Bars = 20 μm. **F** Quantification of total SOD enzymatic activity across the indicated genotypes. Data are presented as mean ± SD (*n* = 3 biologically independent replicates). Statistical significance was assessed using one-way ANOVA followed by Dunnett’s multiple-comparison test, with WT as the control group. *P < 0.05; **P < 0.01. **G** Spatial expression pattern of *ZmCSD5*. RNA in situ hybridization reveals that *ZmCSD5* is specifically expressed within the SAM and developing leaf primordia. Bar = 100 μm. **H** Functional loss of the *ZmCSD5* gene results in a significantly enlarged SAM. Representative images of cleared SAMs from WT and *zmcsd5* mutant. Solid lines indicate the measurement for SAM height and diameter. Bar = 100 μm. **I** Proposed model in which the GRX module restrains ZmCSD5-associated SOD activity, limiting excessive *O*_2_^•−^ scavenging and supporting a localized central-zone superoxide pool and meristem maintenance

To investigate the molecular mechanisms by which *GRX* genes regulate SAM development, we performed RNA-seq analysis on SAMs of 14-DAG WT and *grx* triple mutant seedlings. Transcriptome analysis identified 4,248 differentially expressed genes (DEGs) in the *grx* triple mutant, including 2,714 up-regulated and 1,534 down-regulated genes (Table S2, Figure S1F). Gene Ontology (GO) enrichment analysis revealed that the down-regulated DEGs were predominantly enriched in basic cellular activities and developmental processes (Fig. 1F, Figure S1G), in line with the growth retardation of *grx* triple mutant. Strikingly, the up-regulated DEGs were highly enriched in reactive oxygen species (ROS)-related pathways (Fig. 1G, Figure S1H), suggesting that loss of GRX function triggers severe transcriptional reprogramming of redox-related pathways in the SAM. To characterize the spatiotemporal distribution of ROS homeostasis and its correlation with SAM development, we performed in situ nitroblue tetrazolium (NBT) staining to detect the superoxide anion (*O*_2_^•−^), a major component of ROS, in maize seedlings. In WT SAMs, the NBT signal was enriched in the central zone. The signal was markedly reduced in the *msca1;zmgrx5* double and *grx* triple mutants (Fig. 1H). Quantification of NBT staining intensity confirmed significant reductions in the higher-order mutants, which were accompanied by progressively reduced SAM size (Fig. 1I). To further elucidate the redox status in maintaining meristem activity, we treated seedlings with two broad-spectrum radical scavengers, n-propyl gallate (PG) and N, N'-dimethylthiourea (DMTU) (Fig. 2A). Both treatments with 30 mM PG or 10 mM DMTU reduced SAM size in WT plants and the *grx* mutants (Fig. 2B). These pharmacological data support a general requirement for redox status in SAM development.

To further dissect how GRX proteins maintain superoxide homeostasis in SAM, we analyzed all 37 annotated genes which are likely involved in ROS metabolism (Fig. 2A). By filtering these genes based on existing tissue-specific transcriptomic data (Figure S2) (Han et al. 2023) and subcellular localization prediction (Table S3), further confirming the localizations of selected genes in *Nicotiana benthamiana* (Figure S3), we ultimately selected 16 candidate enzymes for yeast two-hybrid (Y2H) interaction analyses (Figure S4). Yeast two-hybrid screening identified the superoxide dismutases ZmCSD5 and ZmMSD2 as MSCA1-interacting proteins (Figure S4), and these associations were further supported by luciferase complementation imaging and BiFC assays in *Nicotiana benthamiana* (Fig. 2C-E, Figure S5A-C). We prioritized ZmCSD5 for genetic analysis because glutaredoxins (GRX) commonly regulate target proteins through cysteine-dependent thiol modifications. Multiple sequence alignment showed that ZmMSD2 lacks cysteine residues, whereas ZmCSD5 contains two highly conserved cysteines (C119 and C208), providing a testable mechanistic rationale for potential redox regulation (Figure S5D-E). Nevertheless, direct modification of these residues by MSCA1 has not yet been demonstrated. Total SOD activity was significantly elevated in both the *msca1* single and *grx* triple mutants relative to WT (Fig. 2F), supporting a model in which GRX function restrains SOD activity and limits excessive *O*_2_^•−^ scavenging and thereby helps maintain superoxide homeostasis in the SAM.

To clarify the role of *ZmCSD5* in SAM development, we first examined its spatial expression pattern through RNA in situ hybridization. In 14-DAG seedlings, *ZmCSD5* exhibited prominent expression signals throughout the SAM and developing leaf primordia (Fig. 2G). This expression pattern overlaps with the expression domain of three *GRXs* in the SAM (Fig. 1C-E), supporting the biological relevance of their physical interaction. To investigate the impact of *ZmCSD5* on meristem development, we generated several loss-of-function alleles using CRISPR-Cas9. The *zmcsd5-1* and *zmcsd5-2* alleles carry a 20-bp insertion and a 46-bp deletion in the first exon, respectively, both resulting in frameshifts and premature stop codons upstream of the conserved Cu–Zn superoxide dismutase domain (Figure S6A). These mutations likely represent null alleles. Interestingly, the *zmcsd5* mutants exhibited significantly enlarged meristems compared with the WT (Fig. 2H, Figure S6B-C), which contrasts with the reduced-SAM phenotype observed in the *grx* mutants. These findings demonstrate that *ZmCSD5* functions as a negative regulator of shoot apical meristem development in maize.

## Discussion

The shoot apical meristem (SAM), from which all above-ground tissues of plants are derived, is critical to plant morphogenesis and development (Luo et al. 2024). Previous studies have shown that the classical peptide-receptor kinase signaling network (the CLAVATA-WUSCHEL pathway) finely tunes the maintenance and differentiation of the SAM (Somssich et al. 2016). In recent years, reactive oxygen species (ROS) signaling has also been recognized as a critical player in SAM development and homeostasis. However, how active ROS signals are precisely regulated and integrated into SAM developmental network remains to be fully elucidated. In this study, we propose that the GRX module contributes to superoxide homeostasis in the SAM, with MSCA1 physically interacting with ZmCSD5 and potentially restraining ZmCSD5-associated SOD activity.

In both animal and plant systems, ROS have long been regarded as metabolic byproducts or stress signals. However, recent studies indicate that the spatially specific distribution of distinct ROS plays a highly conserved and critical role in animal stem cell self-renewal and plant cell fate determination (Bigarella et al. 2014; Schieber and Chandel 2014). In the maize vegetative SAM, NBT staining revealed a central-zone-enriched signal that was markedly reduced in the *grx* mutants. This pattern is consistent with observations in the *Arabidopsis* SAM (Zeng et al. 2017) and with ROS -based regulation of the root apical meristem (Li et al. 2024), implying that the stem cell niche under the control of ROS spatial gradients is highly conserved across monocots and dicots, as well as in diverse meristem types. PG and DMTU treatments also reduced SAM size, providing pharmacological evidence that redox balance contributes to SAM development. However, because PG and DMTU are broad-spectrum radical scavengers, these pharmacological experiments do not establish a superoxide-specific causal effect. Together, our findings support a role for localized superoxide homeostasis in maize SAM activity and development.

A central biological question in meristem development is how plants actively maintain this highly localized pool of superoxide anions within the stem cell niche. In biological systems, superoxide dismutases (SODs) are critical enzymes that catalyze the rapid dismutation of superoxide anions (*O*_2_^•−^) into hydrogen peroxide (*H*_*2*_*O*_*2*_). Since *O*_2_^•−^ maintains stem cell identity whereas *H*_*2*_*O*_*2*_ generally drives cellular differentiation, excessive SOD activity within the central zone could reduce the stem-cell-associated *O*_2_^•−^ pool and potentially favor differentiation. Our data support a model in which MSCA1 associates with ZmCSD5 and restrains ZmCSD5-associated SOD activity, thereby limiting excessive *O*_2_^•−^ scavenging in the central zone. Consistent with this model, loss of GRX function was accompanied by increased total SOD activity, reduced NBT staining, and decreased SAM size. However, direct biochemical inhibition of ZmCSD5 by MSCA1 and the corresponding effects on *H*_*2*_*O*_*2*_ accumulation remain to be established. Because genetic epistasis between *MSCA1* and *ZmCSD5* has not yet been tested, the genetic hierarchy between these components remains unresolved. Generating *msca1 zmcsd5* double mutants will be important future work.

Together with our previous work about three GRXs in inflorescence development, MSCA1 and its two paralogs have been shown to act as a central hub in the redox regulatory network governing meristem development and maize morphogenesis. During early shoot apical meristem development, the GRX module may contribute to local superoxide homeostasis by restraining SOD activity, potentially through ZmCSD5. During inflorescence meristem development, three GRXs modulate the DNA-binding activity of the key transcription factor FEA4 through direct redox modification, finely tuning the transcriptional network of meristem architecture at the molecular level (Yang et al. 2021). These findings imply that GRX proteins, on the one hand, participate in maintaining superoxide homeostasis by modulating SOD activity, and on the other hand, utilize a specific redox microenvironment to exert their modification on downstream targets such as FEA4. This mode of action, simultaneously intervening in upstream metabolic homeostasis and downstream transcriptional responses, underscores the core roles of these three GRXs in maize development.

In conclusion, our data support a working model in which MSCA1 physically associates with ZmCSD5 and may restrain ZmCSD5-associated SOD activity, thereby limiting excessive scavenging of superoxide anions (*O*_2_^•−^) in the central zone of the SAM and contributing to the maintenance of maize meristem architecture. The discovery of this GRX-SOD redox regulatory module not only elucidates how local superoxide signaling dictates meristem architecture, but also provides novel genetic targets for the molecular improvement of crop yield traits.

## Supplementary Information

Supplementary Material 1.

Supplementary Material 2.

## Data Availability

The RNA sequencing data generated in this study have been deposited in the NCBI BioProject (www.ncbi.nlm.nih.gov/bioproject/) under accession number PRJNA1513733. All other data that support the findings of this study are available from the corresponding authors on request.

## References

[CR1] Andersen SU, Buechel S, Zhao Z, Ljung K, Novák O, Busch W, et al. Requirement of B2-type cyclin-dependent kinases for meristem integrity in *Arabidopsis thaliana*. Plant Cell. 2008;20:88–100. 18223038 10.1105/tpc.107.054676 PMC2254925

[CR2] Barton MK. Twenty years on: the inner workings of the shoot apical meristem, a developmental dynamo. Dev Biol. 2010;341:95–113. 19961843 10.1016/j.ydbio.2009.11.029

[CR3] Bigarella CL, Liang R, Ghaffari S. Stem cells and the impact of ROS signaling. Development. 2014;141:4206–18. 25371358 10.1242/dev.107086 PMC4302918

[CR4] Considine MJ, Foyer CH. Redox regulation of plant development. Antioxid Redox Sign. 2014;21:1305–26. 10.1089/ars.2013.5665 PMC4158970 24180689

[CR5] Cui GC, Li Y, Zheng LY, Smith C, Bevan MW, Li YH. The peptidase DA1 cleaves and destabilizes WUSCHEL to control shoot apical meristem size. Nat Commun. 2024;15:4627. 10.1038/s41467-024-48361-7 PMC11143343 38821962

[CR6] Demesa-Arevalo E, Narasimhan M, Simon R. Intercellular communication in shoot meristems. Annu Rev Plant Biol. 2024;75:319–44. 38424066 10.1146/annurev-arplant-070523-035342

[CR7] Dresselhaus T, Balboni M, Berg L, Dolata A, Hochholdinger F, Huang YY, et al. How meristems shape plant architecture in cereals-Cereal Stem Cell Systems (CSCS) Consortium. Plant Cell. 2025;37:7. 10.1093/plcell/koaf150 PMC12290889 40578304

[CR8] Greb T, Clarenz O, Schäfer E, Müller D, Herrero R, Schmitz G, et al. Molecular analysis of the LATERAL SUPPRESSOR gene in *Arabidopsis* reveals a conserved control mechanism for axillary meristem formation. Gene Dev. 2003;17:1175–87. 12730136 10.1101/gad.260703 PMC196050

[CR9] Han LQ, Zhong WS, Qian J, Jin ML, Tian P, Zhu WC, et al. A multi-omics integrative network map of maize. Nat Genet. 2023;55:144. 36581701 10.1038/s41588-022-01262-1

[CR10] Heidstra R, Sabatini S. Plant and animal stem cells: similar yet different. Nat Rev Mol Cell Bio. 2014;15:301–12. 24755933 10.1038/nrm3790

[CR11] Huang XZ, Chen SD, Li WP, Tang LL, Zhang YQ, Yang N, et al. ROS regulated reversible protein phase separation synchronizes plant flowering. Nat Chem Biol. 2021;17:549–57. 33633378 10.1038/s41589-021-00739-0

[CR12] Huang XZ, Xiao N, Xie Y, Xu C. ROS burst prolongs transcriptional condensation to slow shoot apical meristem maturation and achieve heat-stress resilience in tomato. Dev Cell. 2025;60:2032–45. 40179886 10.1016/j.devcel.2025.03.007

[CR13] Hückelhoven R, Fodor J, Trujillo M, Kogel KH. Barley and mutants compromised in the hypersensitive cell death response against f.sp are modified in their ability to accumulate reactive oxygen intermediates at sites of fungal invasion. Planta. 2000;212:16–24. 10.1007/s004250000385 11219579

[CR14] Jiao CJ, Jiang JL, Li C, Ke LM, Cheng W, Li FM, et al. β-ODAP accumulation could be related to low levels of superoxide anion and hydrogen peroxide in Lathyrus sativus L. Food Chem Toxicol. 2011;49(3):556–62. 10.1016/j.fct.2010.05.054. Epub 2010 May 25. PMID: 20510333. 20510333

[CR15] Li JJ, Zeng J, Tian ZX, Zhao Z, Raikhel N. Root- specific photoreception directs early root development by HY5-regulated ROS balance. P Natl Acad Sci USA. 2024;121–6. 10.1073/pnas.2313092121 PMC10861875 38300870

[CR16] Liu SM, Wu HJ, Zhao Z. Heat stress-induced decapping of mRNA enhances stem cell thermotolerance in *Arabidopsis*. Mol Plant. 2024;17:1820–32. 39468792 10.1016/j.molp.2024.10.011

[CR17] Luo Z, Wu LM, Miao XX, Zhang S, Wei NN, Zhao SY, Shang XY, Hu HY, Xue JQ, Zhang TF, Yang F, Xu ST, Li L. A dynamic regulome of shoot-apical-meristem-related homeobox transcription factors modulates plant architecture in maize. Genome Biol. 2024;25:245. 10.1186/s13059-024-03391-8 PMC11411777 39300560

[CR18] Mayer KF, Schoof H, Haecker A, Lenhard M, Jürgens G, Laux T. Role of WUSCHEL in regulating stem cell fate in the *Arabidopsis* shoot meristem. Cell. 1998;95:805–15. 9865698 10.1016/s0092-8674(00)81703-1

[CR19] Meyer Y, Buchanan BB, Vignols F, Reichheld JP. Thioredoxins and glutaredoxins: unifying elements in redox biology. Annu Rev Genet. 2009;43:335–67. 19691428 10.1146/annurev-genet-102108-134201

[CR20] Mittler R. ROS are good. Trends Plant Sci. 2017;22:11–9. 27666517 10.1016/j.tplants.2016.08.002

[CR21] Rouhier N. Plant glutaredoxins: pivotal players in redox biology and iron-sulphur centre assembly. New Phytol. 2010;186:365–72. 20074091 10.1111/j.1469-8137.2009.03146.x

[CR22] Schieber M, Chandel NS. ROS function in redox signaling and oxidative stress. Curr Biol. 2014;24:R453–62. 24845678 10.1016/j.cub.2014.03.034 PMC4055301

[CR23] Schoof H, Lenhard M, Haecker A, Mayer KFX, Jürgens G, Laux T. The stem cell population of *Arabidopsis* shoot meristems is maintained by a regulatory loop between the CLAVATA and WUSCHEL genes. Cell. 2000;100:635–44. 10761929 10.1016/s0092-8674(00)80700-x

[CR24] Somssich M, Je B, Simon R, Jackson D. CLAVATA-WUSCHEL signaling in the shoot meristem. Development. 2016;143:3238–48. 27624829 10.1242/dev.133645

[CR25] Tsukagoshi H, Busch W, Benfey PN. Transcriptional regulation of ROS controls transition from proliferation to differentiation in the root. Cell. 2010;143:606–16. 21074051 10.1016/j.cell.2010.10.020

[CR26] Voloboeva V, Dequeker B, Van Doorselaer L, Panicucci G, Perata P, Verboven P, et al. The hypoxic niche enclosing the shoot apical meristem is shaped by a combination of morphological features and metabolic activity. Mol Plant. 2026. 10.1016/j.molp.2026.02.011. 41761614 PMC13139043

[CR27] Wang SW, Liu M, Hu DP, Dong ZC, Zhao Z. Control of DNA demethylation by superoxide anion in plant stem cells. Nat Chem Biol. 2025. 10.1038/s41589-024-01737-8. 39266722

[CR28] Weigel D, Jürgens G. Stem cells that make stems. Nature. 2002;415:751–4. 11845197 10.1038/415751a

[CR29] Weits DA, Kunkowska AB, Kamps NCW, Portz KMS, Packbier NK, Venza ZN, et al. An apical hypoxic niche sets the pace of shoot meristem activity. Nature. 2019;569:714. 31092919 10.1038/s41586-019-1203-6

[CR30] Wu QY, Yang J, Cheng NH, Hirschi KD, White FF, Park S. Glutaredoxins in plant development, abiotic stress response, and iron homeostasis: from model organisms to crops. Environ Exp Bot. 2017;139:91–8.

[CR31] Yamada M, Han XW, Benfey PN. RGF1 controls root meristem size through ROS signalling. Nature. 2020;577:85. 31801996 10.1038/s41586-019-1819-6 PMC6930331

[CR32] Yang F, Bui HT, Pautler M, Llaca V, Johnston R, Lee BH, et al. A maize glutaredoxin gene regulates shoot meristem size and phyllotaxy. Plant Cell. 2015;27:121–31. 25616873 10.1105/tpc.114.130393 PMC4330572

[CR33] Yang R, Xu F, Wang YM, Zhong WS, Dong L, Shi YN, et al. Glutaredoxins regulate maize inflorescence meristem development via redox control of TGA transcriptional activity. Nat Plants. 2021;7:1589. 34907313 10.1038/s41477-021-01029-2

[CR34] Zeng J, Dong ZC, Wu HJ, Tian ZX, Zhao Z. Redox regulation of plant stem cell fate. EMBO J. 2017;36:2844–55. 28838936 10.15252/embj.201695955 PMC5623875

[CR35] Zeng J, Zhao XA, Liang Z, Hidalgo I, Gebert M, Fan PF, Wenzl C, Gornik SG, Lohmann JU. Nitric oxide controls shoot meristem activity via regulation of DNA methylation. Nat Commun. 2023;14:8001. 10.1038/s41467-023-43705-1 PMC10696095 38049411

[CR36] Zeng J, Geng X, Zhao Z, Zhou WK. Tipping the balance: the dynamics of stem cell maintenance and stress responses in plant meristems. Curr Opin Plant Biol. 2024. 10.1016/j.pbi.2024.102510. 38266375

