# Supplementary material for: A GRX-SOD module maintains superoxide homeostasis and meristem development in maize
Source: Adv Biotechnol (Singap). 2026 Sep 4;4(3):36. doi: 10.1007/s44307-026-00133-8 (PMC13545189; doi:10.1007/s44307-026-00133-8)
Supplement: Supplementary file 1 [file 44307_2026_133_MOESM2_ESM.pdf]

Figure S1

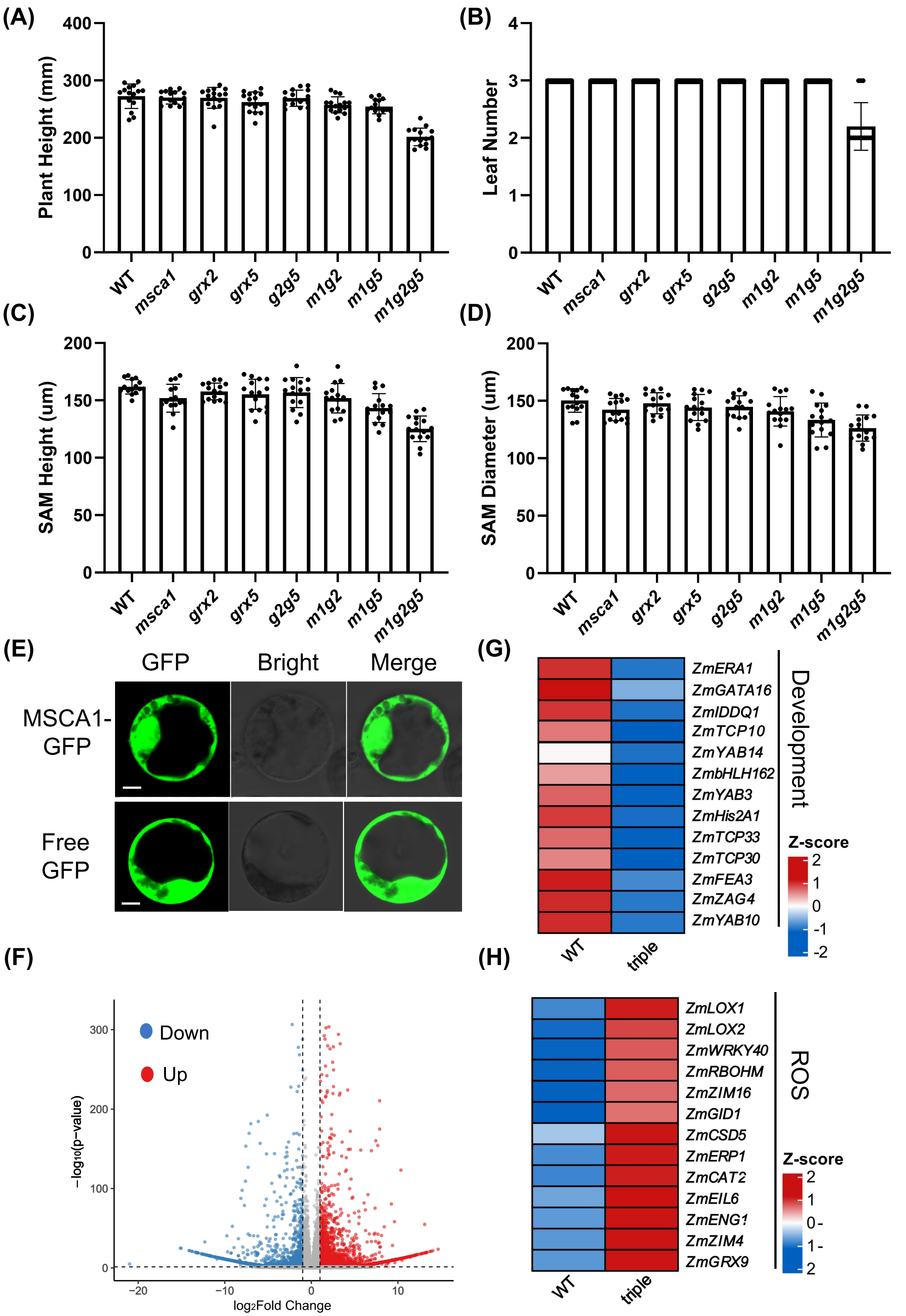

(A)
Plant Height (mm)
0
100
200
300
400
WT
msca1
grx2
grx5
g2g5
m1g2
m1g5
m1g2g5
(B)
Leaf Number
0
1
2
3
4
(C)
SAM Height (um)
0
50
100
150
200
(D)
SAM Diameter (um)
(E)
GFP
Bright
Merge
MSCA1-GFP
Free GFP
(F)
Down
Up
−log10(p−value)
log2Fold Change
−20
−10
0
10
300
200
100
(G)
ZmERA1
ZmGATA16
ZmIDDQ1
ZmTCP10
ZmYAB14
ZmbHLH162
ZmYAB3
ZmHis2A1
ZmTCP33
ZmTCP30
ZmFEA3
ZmZAG4
ZmYAB10
Development
Z-score
2
1
0
-1
-2
WT
triple
(H)
ZmLOX1
ZmLOX2
ZmWRKY40
ZmRBOHM
ZmZIM16
ZmGID1
ZmCSD5
ZmERP1
ZmCAT2
ZmEIL6
ZmENG1
ZmZIM4
ZmGRX9
ROS
Z-score

Figure S1. (A-B) Compared with WT, the *msca1; zmgrx5* double mutants and the *grx* triple mutant exhibit significantly reduced plant height (A). Additionally, the *grx* triple mutant displays a reduced leaf number (B). Data represent average values ± s.d. (n = 15 biologically independent plants). Statistical significance was assessed using one-way ANOVA followed by Dunnett's multiple-comparison test, with WT as the control group. ns, not significant; *$P < 0.05$; **$P < 0.01$; ***$P < 0.001$. **(C-D)** Quantification of SAM height (C) and diameter (D) across different genotypes. A drastic reduction in SAM dimensions was observed in *msca1; zmgrx5* double and *grx* triple mutants. Data represent average values ± s.d. (n = 15 biologically independent plants). Statistical significance was assessed using one-way ANOVA followed by Dunnett's multiple-comparison test, with WT as the control group. ns, not significant; *$P < 0.05$; **$P < 0.01$; ***$P < 0.001$. **(E)** Subcellular localization of MSCA1 in maize protoplasts. Bar = 5 μm. **(F)** Volcano plot of differentially expressed genes (DEGs). **(G-H)** Heatmap showing the expression profiles of representative down-regulated development-related genes (G) and up-regulated ROS-related genes (H) (WT vs. *grx* triple mutant).

Figure S2

		Root	Stem	Leaf	SAM1 (14d)	ear	tassel	Embryo	Endosperm
APXc1.1	Zm00001d047757	656.03	436.20	96.95	738.72	454.68	600.05	984.46	161.90
APXc1.2	Zm00001d028709	1017.49	703.24	204.11	1088.43	627.04	757.95	1810.89	545.29
APXa1	Zm00001d016802	1.25	6.57	62.57	5.58	5.25	5.14	2.67	0.62
APXa2	Zm00001d003643	71.99	45.18	12.62	39.02	48.16	50.99	115.81	68.70
APXa3	Zm00001d023582	57.69	35.36	6.33	41.93	52.65	55.40	48.59	66.04
APXb1	Zm00001d025047	25.28	60.36	0.41	0.15	2.29	3.90	0.02	0.00
APXb2	Zm00001d053223	84.49	89.01	53.62	30.93	49.36	44.01	64.26	125.80
APXc2	Zm00001d007234	297.99	272.44	147.73	506.44	149.10	215.95	121.07	164.82
ZmCATI	Zm00001d027511	0.92	0.23	10.47	0.00	0.00	0.00	0.29	2.05
ZmCAT2	Zm00001d054044	16.98	76.19	55.92	0.82	1.04	22.96	0.92	0.61
ZmCAT3	Zm00001d014848	145.66	102.44	21.15	97.87	98.65	83.63	105.45	58.84
ZmrbohA	Zm00001d023859	30.83	0.05	0.01	0.68	2.17	4.09	17.12	28.56
ZmrbohB	Zm00001d043543	39.58	43.42	8.65	12.28	12.92	14.98	13.60	6.94
ZmrbohC	Zm00001d040974	0.00	0.00	0.00	0.07	0.14	0.05	0.00	0.00
ZmrbohD	Zm00001d038762	64.78	47.78	4.55	14.44	18.21	22.37	17.13	20.73
ZmrbohE	Zm00001d042961	47.39	30.31	1.32	3.10	3.14	3.39	10.77	12.90
ZmrbohF	Zm00001d007426	0.00	0.00	0.00	0.09	0.00	0.03	0.00	0.00
ZmrbohG	Zm00001d032079	4.71	1.82	0.30	0.31	0.77	1.04	0.68	0.42
ZmrbohH	Zm00001d007430	10.42	1.25	0.02	0.48	0.06	0.13	0.08	0.00
ZmrbohI	Zm00001d009248	32.69	78.04	0.99	14.01	21.02	21.54	27.00	4.58
ZmrbohJ	Zm00001d007421	0.00	0.00	0.00	0.10	0.00	0.09	0.00	0.00
ZmrbohK	Zm00001d052653	30.67	29.91	12.67	7.00	7.62	13.50	0.85	1.32
ZmrbohL	Zm00001d009349	0.00	0.00	0.00	0.00	0.09	0.07	0.00	0.00
ZmrbohM	Zm00001d040805	31.22	48.50	0.15	11.16	1.49	1.85	6.17	0.75
ZmCSD1	Zm00001d028232	12.35	17.05	8.75	17.70	19.91	16.28	13.82	21.97
ZmCSD2	Zm00001d029170	287.43	237.14	36.37	284.84	297.04	316.73	142.79	69.07
ZmCSD3	Zm00001d031908	65.67	58.98	13.80	106.11	106.32	92.64	60.11	100.68
ZmCSD4	Zm00001d002611	26.58	21.25	4.91	14.95	11.57	13.00	10.67	16.64
ZmCSD5	Zm00001d022505	251.39	288.45	53.86	113.72	128.80	161.76	141.10	97.51
ZmCSD6	Zm00001d047479	388.24	500.86	57.43	363.99	527.05	506.40	287.98	71.12
ZmFSD1	Zm00001d014632	3.38	4.31	0.65	14.01	17.11	17.29	7.52	5.80
ZmFSD2	Zm00001d036135	10.20	12.01	7.35	15.84	17.65	17.71	38.05	13.99
ZmFSD3	Zm00001d045384	1.30	3.10	0.39	5.82	6.13	5.40	5.69	1.18
ZmFSD4	Zm00001d045538	2.48	3.73	3.73	16.98	16.80	13.05	8.09	21.42
ZmFSD5	Zm00001d025106	3.95	6.49	0.90	13.14	17.79	16.70	9.53	6.04
ZmMSD2	Zm00001d009990	62.98	39.31	5.53	116.83	139.24	158.58	83.64	60.68
ZmMSD1	Zm00001d037859	166.81	159.31	20.19	94.73	160.61	161.50	293.84	290.99

Figure S2. Tissue-specific expression profiles of the major ROS-related genes in maize. Heatmap showing the expression abundance of the major ROS-metabolizing genes across eight different maize tissues (Root, Stem, Leaf, SAM, ear, tassel, Embryo, and Endosperm).

Figure S3

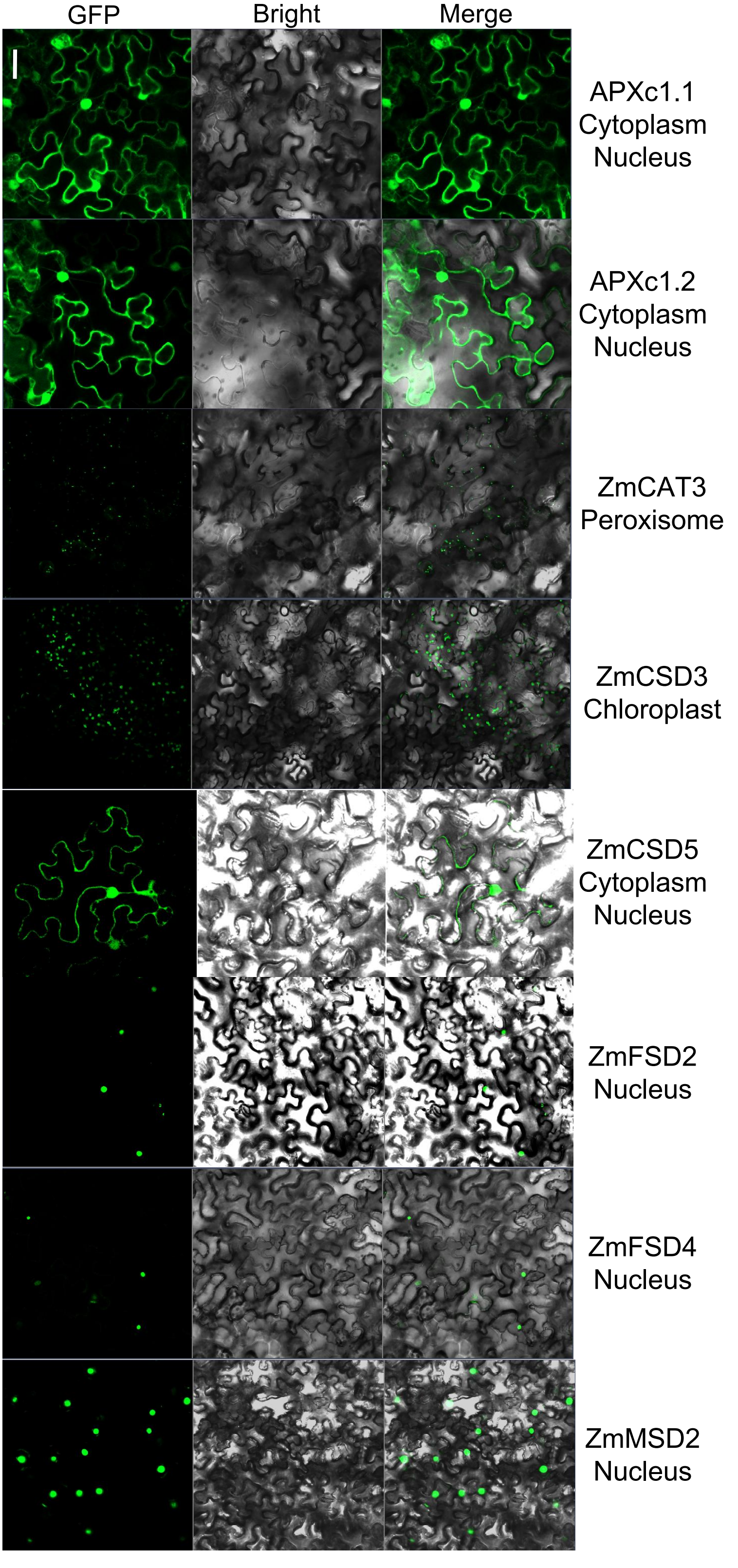

Figure S3 Subcellular localization of selected ROS metabolic enzymes in epidermal cells of *N. benthamiana*. Bars = 20 μm.

Figure S4

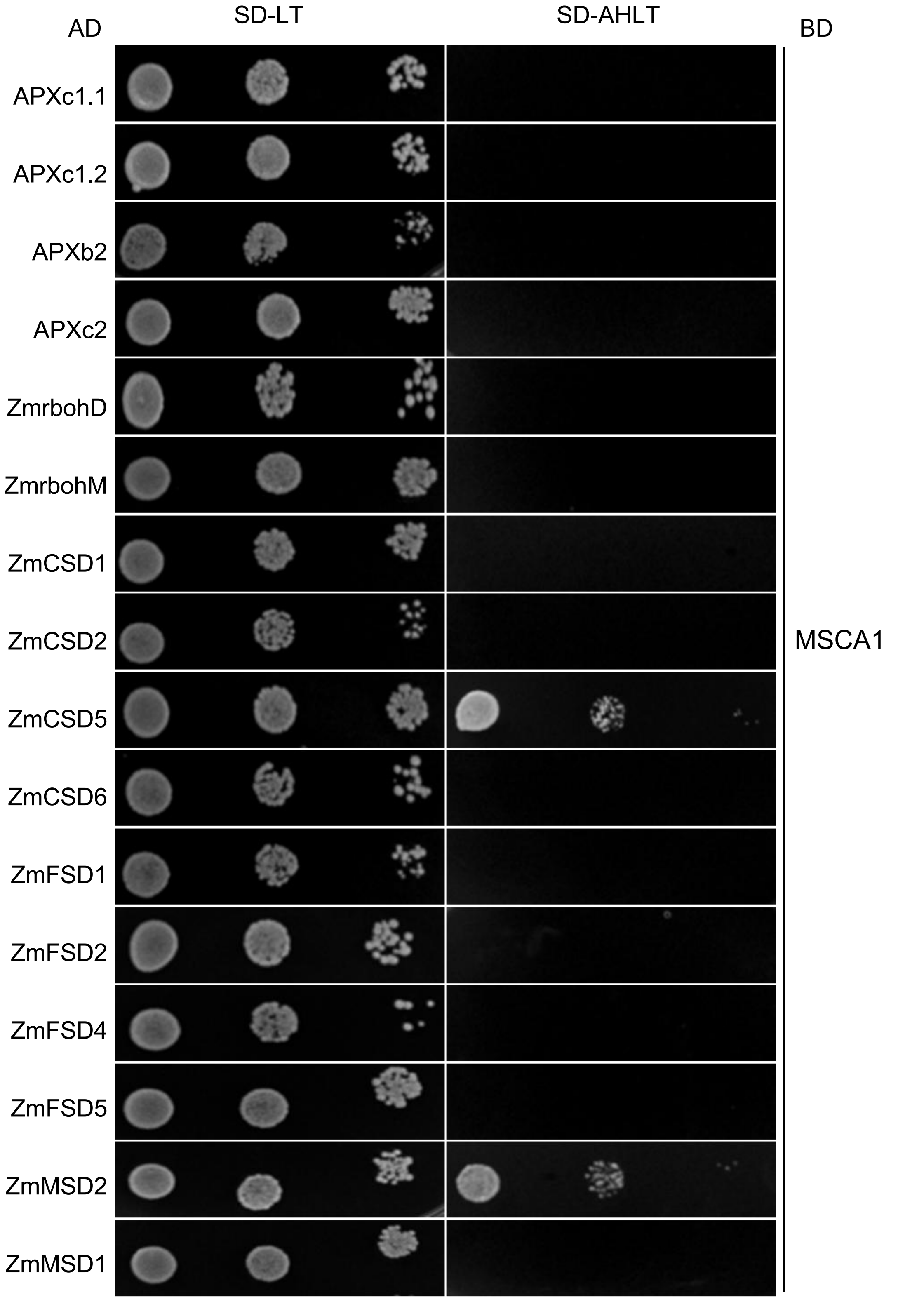

Figure S4. Yeast two-hybrid screening for direct physical interactions between MSCA1 and candidate ROS metabolic enzymes.

Figure S5

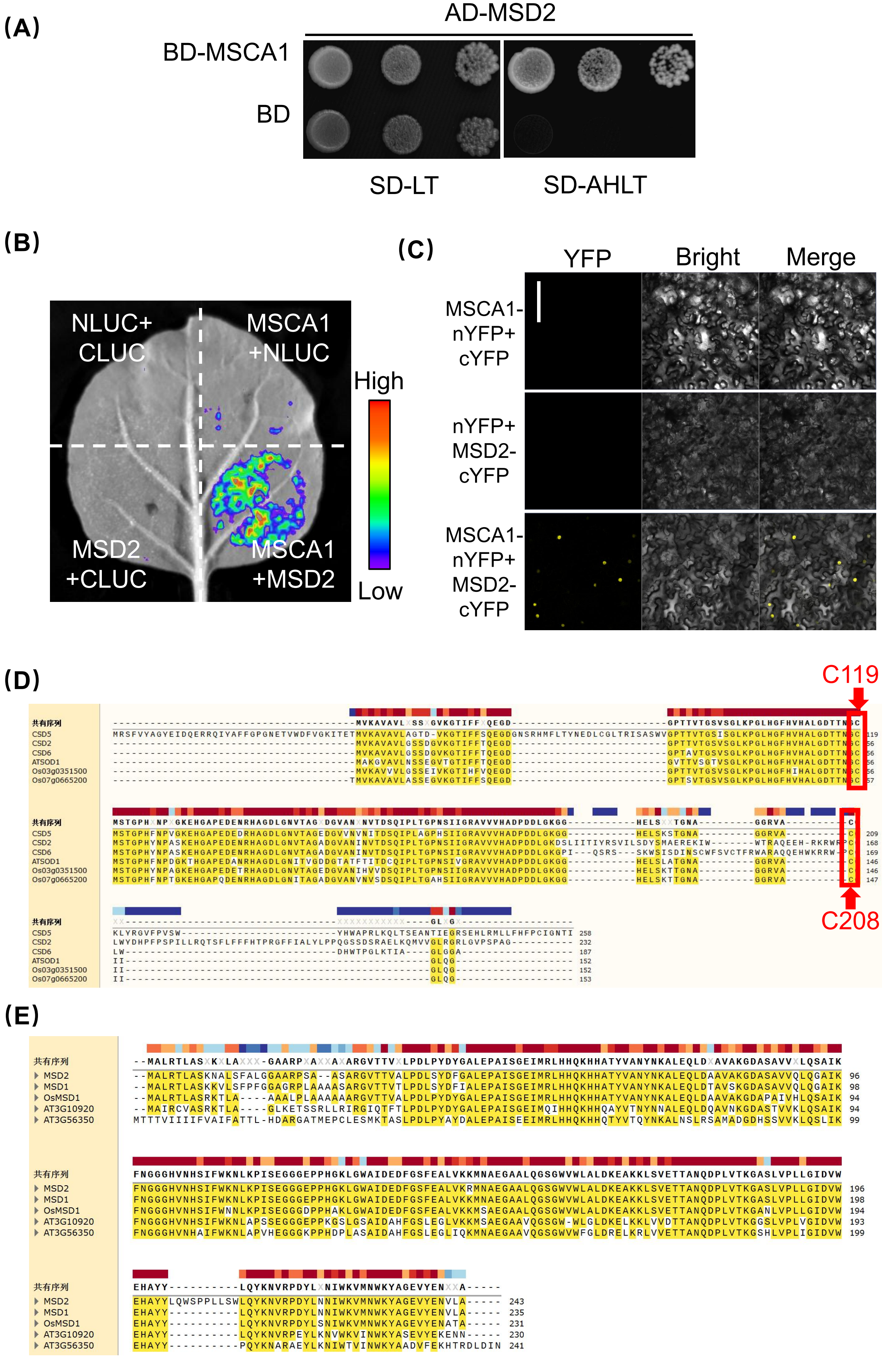
(A)
AD-MSD2
BD-MSCA1
BD
SD-LT
SD-AHLT
(B)
NLUC+
CLUC
MSCA1
+NLUC
MSD2
+CLUC
MSCA1
+MSD2
High
Low
(C)
YFP
Bright
Merge
MSCA1-
nYFP+
cYFP
nYFP+
MSD2-
cYFP
MSCA1-
nYFP+
MSD2-
cYFP
(D)
C119
C208
共有序列
CSD5
CSD2
CSD6
ATSOD1
Os03g0351500
Os07g0665200
(E)
共有序列
MSD2
MSD1
OsMSD1
AT3G10920
AT3G56350

Figure S5. (A) Yeast two-hybrid (Y2H) assays demonstrating the interaction of MSCA1 with ZmMSD2. **(B)** LCI confirmed the interaction of MSCA1 with ZmMSD2. **(C)** BiFC analysis of the interaction between MSCA1 and ZmMSD2 in tobacco leaves. Bars = 20 μm. **(D)** Amino acid sequence alignment of ZmCSD5 with its orthologs shows only Cys119 and Cys208 (red arrowheads) are the conserved cysteine residues. **(E)** Amino acid sequence alignment of ZmMSD2 with its orthologs shows no conserved cysteine residue.

Figure S6

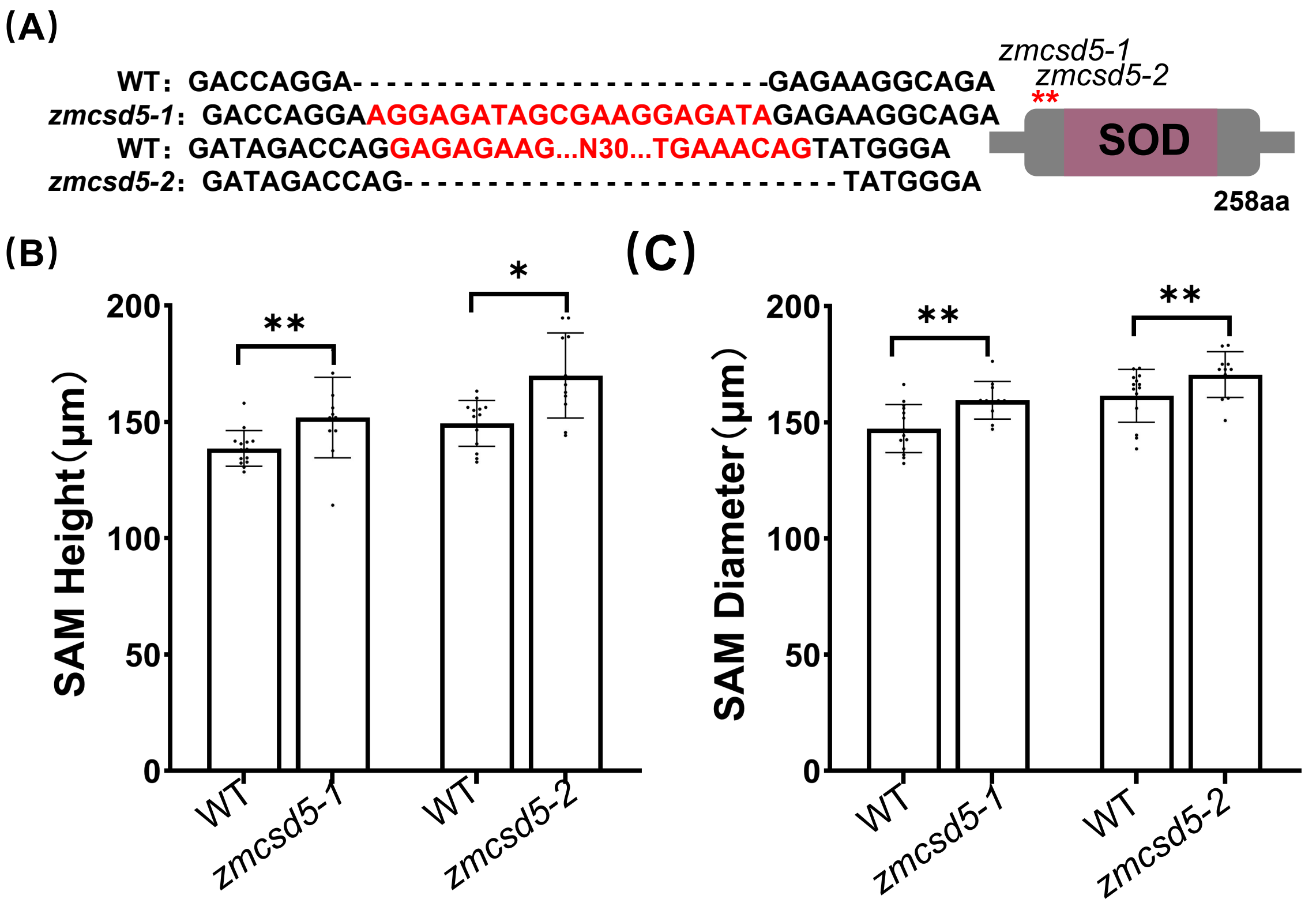

Figure S6. (A) Schematic representation of *ZmCSD5* genome editing using CRISPR/Cas9. **(B-C)** Quantitative assessment of SAM height (B) and diameter (C) across the indicated genotypes, demonstrating the increased dimensions in *zmcsd5* mutants. Data are shown as mean ± SD, dots show data distribution (n = 10 biologically independent replicates, P-values calculated using two-tailed t-test; * $P < 0.05$; ** $P < 0.01$; ***$P < 0.001$).
